# Volitional and Real-Time Control Cursor Based on Eye Movement Decoding Using a Linear Decoding Model

**DOI:** 10.1155/2016/4069790

**Published:** 2016-12-13

**Authors:** Jinhua Zhang, Baozeng Wang, Cheng Zhang, Jun Hong

**Affiliations:** State Key Laboratory for Manufacturing Systems Engineering, School of Mechanical Engineering, Xian Jiaotong University, Xian, 710049, China

## Abstract

The aim of this study is to build a linear decoding model that reveals the relationship between the movement information and the EOG (electrooculogram) data to online control a cursor continuously with blinks and eye pursuit movements. First of all, a blink detection method is proposed to reject a voluntary single eye blink or double-blink information from EOG. Then, a linear decoding model of time series is developed to predict the position of gaze, and the model parameters are calibrated by the RLS (Recursive Least Square) algorithm; besides, the assessment of decoding accuracy is assessed through cross-validation procedure. Additionally, the subsection processing, increment control, and online calibration are presented to realize the online control. Finally, the technology is applied to the volitional and online control of a cursor to hit the multiple predefined targets. Experimental results show that the blink detection algorithm performs well with the voluntary blink detection rate over 95%. Through combining the merits of blinks and smooth pursuit movements, the movement information of eyes can be decoded in good conformity with the average Pearson correlation coefficient which is up to 0.9592, and all signal-to-noise ratios are greater than 0. The novel system allows people to successfully and economically control a cursor online with a hit rate of 98%.

## 1. Introduction

Recently, there has been a wide variety of applications on bioelectricity of the human with the development of HCI (human-computer-interaction) [1]. The EOG (electrooculogram), which is simplest bioelectrical signal, is recorded by skin electrodes placed on the skin around the eyes to detect the eye movements of a stationary subject by measuring the voltage difference. Because of the high SNR (signal-to-noise ratio), the EOG has been properly applied into the field of medical rehabilitation and has drawn the attention of a wide range of researchers due to the great application prospects [2, 3].

Mainly, a large number of researchers from the field of EOG-based HCI focus on two different approaches. The first one studies pattern recognition [4, 5]. There are two main kinds of eye movements, namely, saccade and smooth pursuit movements. The first one is rapid eye movements that align the fovea with the target, but the second one is much slower tracking movements of the eyes designed to keep a moving target on the fovea, which means that it directs the eyes to follow a moving visual target [6–9]. However, there are a lot of restrictions in complex control for less information of the pattern recognition. Then, some researchers designed some predefined sequences of saccades to increase the control patterns [10]. Thus, linear saccadic eye model and eye blink were analyzed using wavelet transformation and fuzzy logic to classify different eye movements in real-time [11]. The second researches on the model of the mutual relationship between eye movements and EOG. Lv et al. detected the relationship between EOG information and eye blink or eye movements to categorize different types of eye movement into four commands which controlled the computer or other instruments [12]. Barea et al. proposed an advanced model based on wavelet transform and neural networks to determine the eye movements and position in terms of the recorded EOG [13]. But the difference is that Tokushige et al. turned on at a random location 5°, 10°, 20°, or 30° horizontally to the left or right [14]. Thus, the system was able to detect movement angles but could not improve the accuracy of the decoded EOG trajectory. However, a linear model was designed and built using eye saccade angles and EOG based on linear fitting by He et al. [15], while Estrany et al. established a similar model using multiple linear regression [16, 17]. In the research of Estrany et al., the adjusting of proportion and excursion parameters, multichannel EOG could be transformed into cursor positions [18]. Tecce et al. represented a moving fixation point on a computer display to select letters by controlling a cursor with eye movements [19]. However, the transformation system is substantial complexity, and subjects are required to spend a long training time carrying out a number of experiments to set the parameters.

Then, there are few investigations on real mapping of the relationship between EOG and the eye fixation positions information directly. Furthermore, there also exist a few issues that remain concerned with online control in the current study, for example, a large range of individual differences, line noises, and baseline artifacts. Therefore, whether the observed object movement information can be decoded from continuous EOG direct and efficient online control is an open question, and if it is possible, there will be a novel system for a real-time interactive control between subject and cursor based on eye movement conveniently. This is the starting point of our research. So this study presents a time series-based decoding model using linear filtering to predict the object motion information in the time domain from EOG. The model parameters can be determined by a simple calibration procedure. Then, a series of processing methods are brought forward to produce online decoding and freely online control cursor.

## 2. Materials and Methods

### 2.1. Participants

Five subjects (2 male and 3 female, age from 22 to 26) of Xi'an Jiaotong University with no relevant diseases participated in this study. All of them provided written informed consent and experimental protocol by Xi'an Human Subject Review Committee before the experiments.

### 2.2. Experimental Condition and Procedure

#### 2.2.1. Experimental Condition

Two experiments were carried out in a brain computer interface laboratory at a fixed time during regular working hours. Subjects were asked to sit in front of a 24-inch flat screen with a resolution of 1,920 pixels 1,200 pixels, with their eyes looking squarely at the horizontal center of the screen which was presented in a black ground. A Neuroscan NuAmps Express system (Compumedics Ltd., VIC, Australia), which collected a wide variety of multichannel neurophysiological signals such as EEG, ECG, EOG, and EMG, was used to record EOG at a sampling rate of 500 Hz. Besides, a notch filter was normally used to remove 50 Hz.

**Figure 1:**
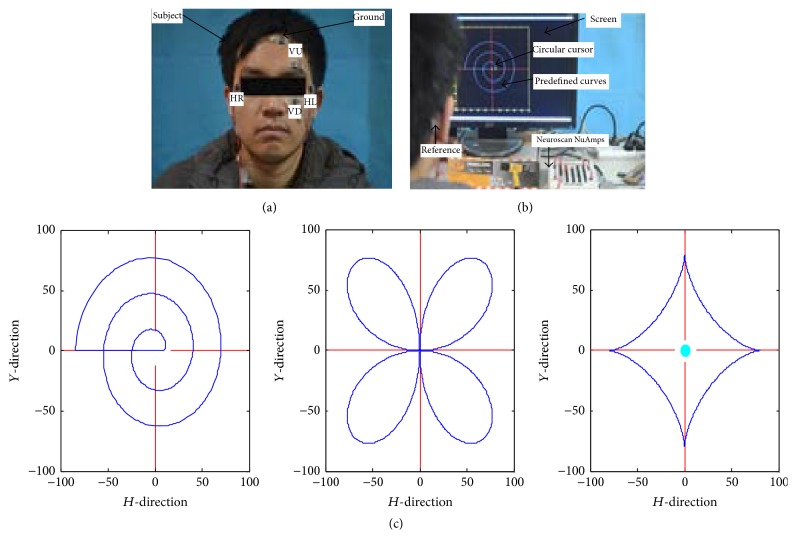
(a) Skin electrodes placement in EOG acquisition; (b) calibration experiments for a helix pattern; (c) experimental patterns: helix pattern, clover pattern, and stellate pattern.

Two pairs of skin electrodes are placed on the opposite sides around the eye. One pair of electrodes is arranged to record the EOG of the right horizontal (HR) and the left horizontal (HL) positions, respectively, and the other pair is placed on the left eye and is used for recording the EOG of the up vertical (VU) and the down vertical (VD) positions separately. In addition, a ground electrode for EOG recordings was attached to the forehead and the mastoid behind the right ear is chosen as reference electrode in Figure 1(a). The HEOG and VEOG can be acquired by the electrodes of the horizontal and vertical directions. So, the different values between two channels in each direction are computed based on the EOG of two pairs of electrodes in

(1) UHt=UHRt−UHLtUVt=UVUt−UVDt,

where *U*_*H*_ is the HEOG (horizontal EOG); *U*_*V*_ is the VEOG (vertical EOG); *U*_HR_, *U*_HL_, *U*_VU_, and *U*_VD_ are the EOG collected by skin electrodes of HR (HEOR), HL (HEOL), VU (VEOU), and VD (VEOD), separately.

#### 2.2.2. Experimental Procedure

All subjects are instructed to track the circular cursor with their eyes, no matter whether it is moving or static, with their bodies (and especially their heads) still and their eyes blinking naturally. But there is sometimes not a moving target which directs the eyes to move in online cursor control. When the eyes quickly moves, the eye movements may be a saccade, and then the eyes slowly move; the eye movements may be smooth pursuit movements. So the eye movements may be called eye pursuit movements which are a hybrid of those two approaches in this study. Subjects take 10 minutes to familiarize themselves with the experimental procedure. The experiment includes calibration and online control cursor. The first one can be seen in Figure 1(b), where participants track the circular cursor which moves smoothly along predefined curves on the screen and the EOG is recorded synchronously. In Figure 1(c), a helix pattern, a clover pattern, and a stellate pattern are designed to evaluate assessment of decoding accuracy. Each pattern is performed five times for each subject. The second one is shown in Figure 7(a): subject online controls the cursor using eye pursuit movements without any trip trajectory. The black interface is divided into eight target areas in the screen edge and one buffer zone in the center. The red circular cursor is initialized at the interface center. Subjects control online and change the cursor position one by one based on eye pursuit movements. When the cursor hits one of the eight target areas, the area becomes yellow, signifying the end of a trial. Then, the cursor can be reset to the center of the screen using a single blink, and in the meantime the vision focus of a subject is set back to the center to begin the next trial. The subjects control the cursor to hit the predefined target areas one by one; the hit times and hit rate are recorded calculated simultaneously.

#### 2.2.3. Signal Acquisition

The pretreatment is the first step before any further analyses. Since the EOG have been recorded by a DC amplifier, DC offsets and drifting can occur artificially and simultaneously. The DC offset is a constant and steady offset from zero voltage, while the DC drifting is a gradual shift that may occur throughout the recording period. The offset and drift together can be merged into a baseline. There are few baseline removal methods for EOG in literature. To avoid the unnecessary DC drift problem, many researchers adopt AC-coupled amplifications or process the signals using high-pass filtering. However, this method also removes the DC component of EOG themselves. Thus, we can not observe the gradually changing information completely. Here, a low-order polynomial fitting method [20], in which the low-order polynomial represents the slow change of the signal, is used to process offline EOG data. A high polynomial order does not mean a high quality but might allow us to extract more detailed information from the EOG. In offline baseline removal, because of the long length of the data, the polynomial order is 3. Subtracting the baseline from the original EOG in each channel allows the signal to start from zero.

As mentioned above, EOG contains noise from different sources. Three methods are widely used to combat this: low-pass filtering, wavelet transforms based on a threshold [21], and median filtering [4]. The median filter of three methods work well enough for artifact rejection. When there are different scanning patterns, the filter holds the edge steepness, which retains the EOG amplitudes and prevents introduction any artificial signal effectively. Furthermore, the median filter is conducted wholly in the time domain, which is good for online control. Considering all the above, a median filter with 150 ms window size is adopted to denoise the EOG.

### 2.3. The Method of Blink Detection

An eye blink associates with a great activity from the human eyes but does not include information about the movements of the observed objects. Thus, an eye blink, which needs to be processed firstly, affects the EOG in the vertical direction with a peaked pulse. Both the voluntary and involuntary blinks appear during blink activity; the voluntary blinks can be used in control, while the involuntary blinks need to be removed for their uncontrollability. The general rule is that an involuntary blink has lower amplitude than a voluntary blink because of its smaller motion. Our experimental statistics results also show that different types of blinks were usually distributed in different voltage ranges. With this in mind, we propose a new blink detection method. After removing low-frequency components of EOG, the method firstly performs the threshold processing twice to obtain the positions of voluntary and involuntary blinks.

**Figure 2:**
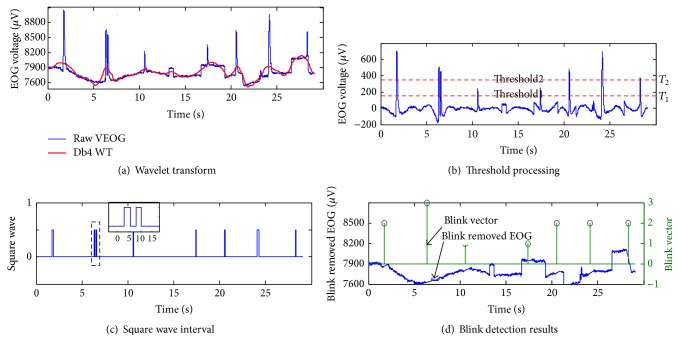
Example of blink detection process. (a) VEOG and low-frequency approximations after wavelet transform; (b) threshold processing; (c) square waves; (d) blink-removed VEOG and separated voluntary blink. In blink vector, a value of 1 is an involuntary blink, 2 is a voluntary single blink, and 3 is a voluntary double-blink.

The blink detection process is shown in Figure 2; the low frequency of raw EOG data *U*_Va_ are extracted by a 1D discrete wavelet decomposition at a 10-detail level using a 4th-order Daubechies as a mother wavelet. After reconstructing the high-scale low-frequency approximations, the trend becomes clear in Figure 2(a). The first threshold processing of the *U*_Vd_ is performed. In Figure 2(b), if the signal is larger than the threshold *T*_1_, it will be set to 1; otherwise it will be set to 0. Then, the signals become square signals which are shown in Figure 2(c). Thus, the second threshold processing is performed on the blink peak values, that is, *U*_Vd_. If the value is larger than the threshold *T*_2_, the blink will be defined as a voluntary blink. Otherwise, it is defined as an involuntary blink. To increase the control effectiveness and complexity, the distances between adjacent single voluntary blinks must be computed. If they are smaller than the blink intervals, the two fast continuous voluntary blinks will be taken as a double-blink. The two blinks will be removed to ensure that only single blinks are saved in it. The first positions of the two blinks will be defined as the double-blink position and will be saved. So far the blink positions are detected; meanwhile, the voluntary single and double-blinks are separated as shown in Figure 2(d).

Generally, the duration of a single blink ranges from 100 to 400 ms [22]. To remove the effects of the blink on the signals, we estimate the precise blink intervals and perform linear interpolation compensation to the intervals. For involuntary blinks and voluntary single blinks, we take the regions from 100 ms before to 250 ms after the blink positions to form the precise blink intervals. For double-blinks, we take regions from 100 ms before the double-blink positions to 500 ms after as the double-blink intervals. By linearly inserting the same number of points into the estimated blink intervals, blink-removed vertical EOG can be produced.

### 2.4. Linear Decoding Model

#### 2.4.1. Construction of Linear Decoding Model

Many researchers have successfully estimated the limb kinematical parameters of humans or animals from neuronal action potentials in recent years. By using bioelectrical signals from the skin to decode the movement information form neuronal activity of the body, the intentions or the limb position feedback of subjects can be connected with the features of the task, and neural decoding models can be built. One of the most widely used models was the linear decoding model [23–25], which used a multiple linear regression or a wiener filter, using the weighted linear combination of neural activities before the current decoding time point to establish the linear decoding model.

Most EOG research only considers the signal from the current time point, ignoring time series effects. Therefore, the linear decoding model is illustrated to extract movement information from EOG. The linear models are described as follows:

(2) ht=a0+∑k=1n−1akUHt−k+εhtvt=b0+∑k=1n−1bkUVt−k+εvt,

where *h*(*t*), *v*(*t*) is a set of both directions (horizontal and vertical directions) through decoding trajectories from EOG at time *t* separately; *n* is the number of lags or the order of the model; here *n* is 10; *U*_*H*_(*t* − *k*), *U*_*V*_(*t* − *k*) is a set of preprocessed and blink-removed EOG in both directions at time *t* − *k*, respectively; *a*_*k*_, *b*_*k*_ is a set of coefficients; *a*_0_, *b*_0_ is the set of intercepts (the value of *h*(*t*) and *v*(*t*) when *U*_*H*_(*t* − *k*) = 0 and *U*_*V*_(*t* − *k*) = 0); *ε*_*h*_, *ε*_*v*_ are the residual errors in both directions.

#### 2.4.2. Parameter Calibration Based on the RLS Algorithm

The linear decoding model above is an *N*th order AR model (autoregressive model), and *a* and *b* of the parameters are calibrated by EOG data. An adaptive filter, which is commonly used in system identification, can revise and update the parameters to satisfy the performance requirements. Furthermore, The EOG is time-varying and nonrepetitive, thus, the adaptive filter algorithm can be used to train the parameters. The RLS algorithm is famous for its fast convergence. The calibration is performed in both the horizontal and vertical directions. The inputs and the ideal outputs of the RLS algorithm are EOG and the real moving object trajectories, respectively. The algorithm order is equal to the model order. Details of the RLS algorithm could be seen in related literatures [26, 27]. After the training is finished, the model parameters can be determined.

#### 2.4.3. Assessment of Linear Decoding Model

For assessment of linear decoding model, three kinds of experimental patterns were designed to test decoding accuracy which reflected the decoding performance or prevented a single pattern from leading to excessive training that brought in this universal adaptability degradation. For individual subject, three EOG data sets of each pattern are averaged into one data set to obtain more steady data set of the training, which are used to adopt cross-validation method to determine the model parameters after blink removal and preprocessing. Then, the cross-validation is performed cross subject and cross pattern to test the accuracy of the decoding models. Respectively for each subject, the RLS Algorithm, which is employed for training the EOG data in one pattern and the desired trajectory, is very helpful to select the most appropriate predicting model. Then, the decoding model is used to decode motion trajectory using one of the three EOG data sets of the other two patterns. Thus, each pattern is used for training once and testing sixth.

The decoding performance of the trained model is assessed by statistical analysis of SNR and *R* (Pearson correlation coefficient) between the known object positions and the object ones reconstructed from EOG [25]. The SNR is defined as the ratio of the power of the EOG to the noise power, that is, the ratio of the squared amplitude of EOG to the amplitude of noise. So the SNR can be calculated from the following equation:

(3) $$\begin{array}{c} \text{SNR}\left(\;x,\hat{x}\;\right)=10\;\log_{10}\left[\;\frac{\text{Var}\left(x\right)}{\text{MSE}}\;\right], \end{array}$$

where *x* is the amplitude of EOG; $\left(\hat{x}\right)$ is the difference between predicted signal and actual ones; Var(*x*) is the variance of the trajectory coordinates; $\text{MSE}(\hat{x})$ is the mean squared error of the predicted signal; $\text{SNR}(x,\hat{x}$) is the ratio between Var(*x*) and $\text{MSE}(\hat{x})$;

The $\text{SNR}(x,\hat{x}$) is converted into a decibel (dB) scale, which means the noise signals present in the reconstructed trajectories. For instance, a SNR of reconstructed EOG trajectories is less than 0, which means weak signal decoding ability that indicates a noisy reconstruction. Then, the SNR is more than 0, which is strong signal decoding capability that stands for a high quality reconstruction EOG decoding trajectories.

In addition, *R* can be defined as the strength of a linear association between the known trajectory and the output of the trained model by the following adaptive RLS algorithm. So the formula of *R* is shown as follows:

(4) $$\begin{array}{c} R\left(\;x,\hat{x}\;\right)=\frac{\text{cov}\left(x,\hat{x}\right)}{\sigma_{x}\sigma_{\hat{x}}}, \end{array}$$

where *x* is the actual trajectory coordinates followed by the eye gaze object; $\hat{x}$ is the predicted output of the model; *σ*_*x*_ and $\sigma_{\hat{x}}$ are the standard deviations of *x* and $\hat{x}$, respectively; *R* is the value ranges from −1 to 1.

From (4), we can infer that the value of *R* is close to 1; it means the highest possible correlation between the actual and decoded trajectories, and the values are close to −1; it presents an inverse relationship, but when the value reaches 0, it indicates the absence of a correlation.

### 2.5. Online Control Cursor

#### 2.5.1. Online Processing

The program of the acquisition system is provided to read the recorded data online; then real-time control is produced through self-developed online processing and control algorithms, so the program reads a sample block at each time point and saves the data in both horizontal and vertical directions. The sample frequency is 500 Hz, and every second has 25 sample blocks, so 20 points are contained in one sample block for each channel. In performing online control, there are several issues with decoding and control. First, because of subject's individual difference, the effects of decoding and control are not satisfactory for different subjects and different conditions. Second, the detection method works well only when the blink activities are successfully identified, especially for a double-blinks. Third, the method outlined above based on low-order polynomial fitting is appropriate for the offline processing of a long data set, not for the online handling of a short data set in baseline removal. Then, the measures of online calibration, subsection processing, and incremental control need to be taken to solve these problems.

**Figure 3:**
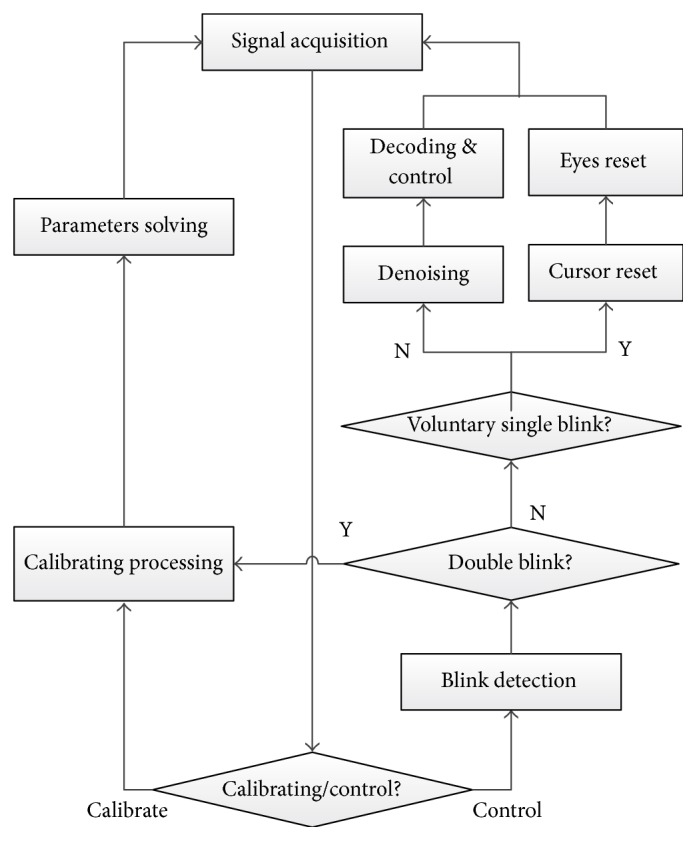
The flow chart of whole online process.

The whole online processing process is shown in Figure 3; the program reads and updates the acquired EOG data. If subjects are running the control program for the first time or need recalibrating, the calibration process can be executed and the model parameters also are updated. After that, the control program is started. It first performs blink detection. After a long time running, if the program breaks, users can restart the calibration process by double-blinking. If there is no double-blink, the system judges whether there is one voluntary blink. If there is, the cursor is reset to the screen center; meanwhile, the subjects eyes also have to go back to the center to perform the next control movement. If not, after denoising by the median filter, the decoding process continues to control the cursor position, so the subsection processing and increment control still need to be carried out. The control results can be fed back in real-time via viewing the screen of PC based on eye movement decoding for a single-trial, so subject performs the next movement by smooth pursuit movements.

#### 2.5.2. Online Calibration

Before the start of control or when the system needs re-calibrating, a calibration dialog pops up, showing the calibration procedure and the system begins saving the EOG data in responsefile.txt simultaneously. The subjects initially face the center of the screen with no head movement. If there are 5 seconds of cease, the cursor slowly moves on the screen along the predefined trajectory, and the subject have to smoothly follow the cursor with smooth pursuit movements. When the trajectory finishes, there are another 5 seconds, the calibration dialog disappears automatically, and EOG data are stopped saving. Then, the parameters of the linear model are solved and saved by the RLS algorithm.

#### 2.5.3. Subsection Processing

**Figure 4:**
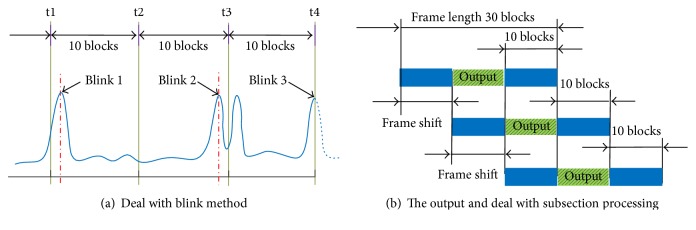
Subsection processing.

Each blink should be in each subsection data for online subsection processing. Thus, as shown in Figure 4(a), blink 2 and blink 3 exhibit two subsections of data, especially for a double-blink where one blink is in the one subsegment, and the other is the next one. This situation often causes great trouble to be detected correctly. So, a number of measures should be taken to improve the detection accuracy. Then, in Figure 4(b) the system always saves the newest 30 blocks which are divided equally into three subsections, that is, ten blocks for each subsection. But every time 10 new sample blocks are read at the next 5 seconds. So, every three subsections are detected and decoded to output the control results of the middle subsection. After that, the program continues to read and update data until another new 10 blocks which are read for the next processing subsession. The control results are continuously decoded without any loss of EOG data.

It may be that convenient to reduce the output and control frequency; then, the average decoding position on the middle subsection is taken as the output for this time point. So the result shows that the interval between two subsection continuous outputs is less than 0.5 s, and the time delay of the control is less than 1 s, and time delay of control output is about 0.8 s, which is the time of update 20 sample blocks.

#### 2.5.4. Increment Control

**Figure 5:**
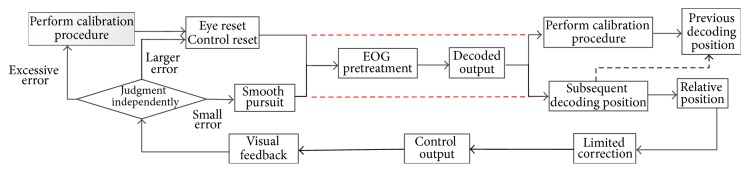
The schematic diagram of online drift correction based on relative coordinates.

The directly decoding method comes within the absolute coordinates, so it is easy to accumulate errors, without any adjustment or flexibility. Thus, the incremental control comes forward to eliminate for accumulate errors, that is to say the control of relative coordinates is adopted to online control cursor based on relative coordinates. In Figure 5, initially, subjects have to stare at the screen center as mentioned before without strabismus. The controlled circular cursor is also initialized in the center of the screen. The previous output position for this subsection is subtracted from the current one to get a relative change or relative coordinates. The system updates the new cursor position according to the current position and the relative change in coordinates. The first output position is subtracted from itself; that is, it stays still at the center of the screen. To reduce errors, if the relative position change exceeds a preset threshold, it is set as the threshold value. The human eye coordinates also have to be transformed into screen coordinates to control the cursor. When there is a small error, the subject still controls the cursor through smooth pursuit movements. Once the error is larger, the subject has a voluntary single blink to reset reference point to keep consistent in fixation point coordinates and control coordinates, so the accumulated errors can be eliminated. Furthermore, if the error is excessive, the system needs recalibrating. The online drift correction efficiently eliminates the impact of baseline drift and improves the control flexibility.

## 3. Experimental Results

### 3.1. Blink Detection

According to the experimental statistics, when the threshold *T*_1_ is set to 80 *μ*V, all blinks easily are detected. However, the threshold *T*_2_ has a great effect on the results of the detection of the separation of voluntary and involuntary blinks. Blink EOG data are used to train the threshold *T*_2_. We set *T*_2_ values from 300 to 700 *μ*V with every 5 values to take a value and obtained 81 values in total. The EOG data used for training lasted about 16.3 minutes and contained 470 blinks. The optimal threshold is 515 *μ*V according to ROC curve. The threshold is used to process another data set that lasts 16.8 minutes and contains 480 blinks. The accuracy rate of blink detection is 96.88%, with 6 false detections and 9 missing detections.

Because the responses of false detections may cause large potential security problems, while missing detections have no effect on control. In real HCI, it is thus better to increase the optimal threshold value appropriately to reduce false detections. Based on this, the final chosen threshold is increased to 540 *μ*V. The effect of the new threshold on the detection results from the test data is 3 false detections and 21 missing detections, with a new accuracy rate of 95.00%. So the threshold value is set at 540 *μ*V in the end to reduce the number of false detections.

### 3.2. Linear Decoding

The best value is subject 3 and the worst value is subject 5 in the average values and standard deviations of the SNR and *R*; then the mean values of five subjects are shown in Table 1. In the horizontal data for all subjects, for the SNR the minimum is 8.95 and the average is 17.95, and for *R* the minimum is 0.9777 and the average is 0.9943. In the vertical data for all subjects, for the SNR the minimum is 1.39 and the average is 11.57, and for *R* the minimum is 0.7536 and the average is 0.9592. Overall, correlation values across the subjects are higher in the horizontal than in the vertical direction. All of these data confirm good decoding performance with the average *R* > 0.9592 and high SNR (all > 0).

**Figure 6:**
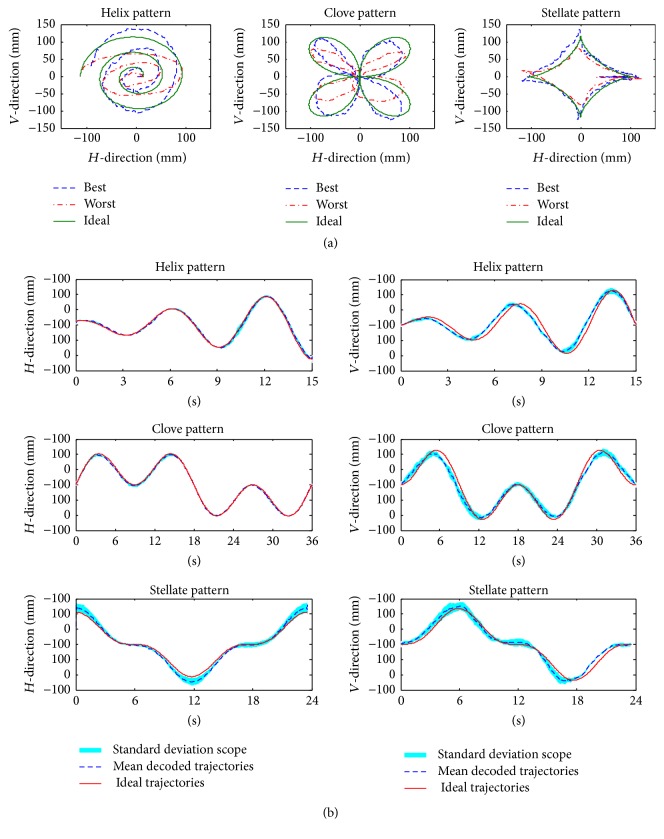
(a) The best and the worst reconstructed trajectories in 2D space; (b) ideal trajectories, mean decoded trajectories, and their standard deviations.

Figure 6(a) shows the best and the worst reconstructed trajectories in 2D space for all decoded results. It is easy to see that the decoded trajectories retained the movement information of the ideal trajectories, and the goodness of fit is higher for the simpler patterns. The decoded trajectories of all the subjects are analyzed statistically, and the mean trajectories and their standard deviations are shown in Figure 6(b). For the error between the mean decoded trajectories and the ideal trajectories for all the patterns, the average is 4.17 mm (the maximum is 9.31 mm) and 15.32 mm (the maximum is 29.77 mm) in the horizontal direction and the vertical direction, respectively. Thus, the decoding model could be used for some simple and continuous control, such as a simple interface with a cursor on a PC.

The EOG decoding accuracy in the vertical direction is not as good as the horizontal, especially for a large-scale smooth pursuit movements. The main reason is that the vertical EOG is much more prone to being affected by EMG from sources like frowns or other facial expressions, which occur randomly with varying degrees. The experiments show that even a slight frown or facial expression can cause large signal changes. In addition, EOG with more blinks deviated heavily, even with a blink detection method, linear insertion cannot completely compensate for these effects. All of these factors will influence the processing results.

### 3.3. Online Cursor Control

The EOG data have been processed with data block partition based on online subsection processing. The program receives a sample block every 40 ms and every update 10 blocks output the control result, so the position control frequency can be up to 2.5 Hz based on eye pursuit movements; that is, the target position can be changed by means of eye movements every 2 s. However, the target position changes do not directly output for controlling, the cursor enters and come out corresponding area to achieve control result, so the control frequency is lower than position control one based on eye pursuit movements. Although the control frequency slightly decreased, the subject acquires the current position feedback according to the cursor's location; thus, each subject has greatly increased a certain capacity of self-adaptation.

**Figure 7:**
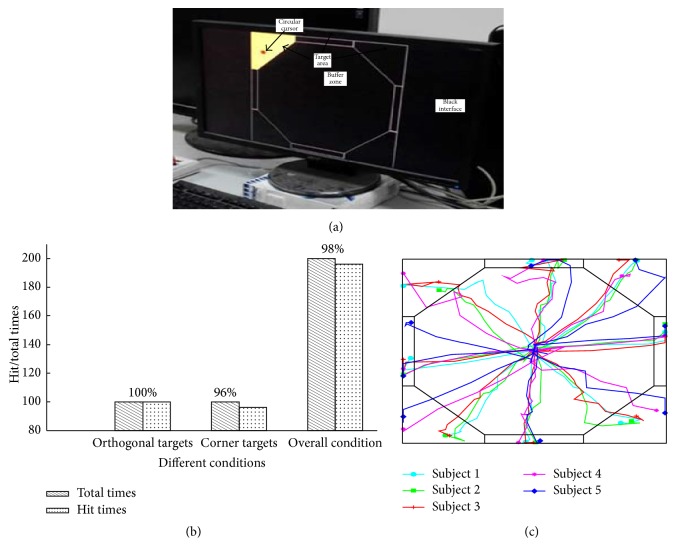
(a) Cursor Control Interface; (b) hit rates of the cursor control; (c) one cursor control trajectory for each participant.

Each subject controls the cursor on the screen by the eye pursuit movements that slowly change the point of fixation to hit the predefined target area one after another. Meanwhile, the trajectories of cursor movement are recorded and saved. The cursor control trials are carried out five times for each subject and the experimental results can be seen in Figure 7(b). There are two different conditions in the histogram. One is four orthogonal targets (top, bottom, left, and right). Each subject hit 100 times in 100 times, so hit rate is 100%. Then the other is four corner targets (left top, left bottom, right top, and right bottom) whose hit rate on average is 96%. From the hit rate of two different conditions, it is easier to hit the targets in the orthogonal directions than the corner one. At last, the rate for overall hit is 98%. One of the four control results for each subject is selected randomly, and the drawn results of cursor movement trajectories are presented in Figure 7(c).

## 4. Discussion

In this study, subjects are asked to perform volitional and real-time control cursor of an interface using voluntary single eye blink and eye pursuit movements. The new system performs well with the voluntary blink detection rate over 96.88% with 6 false detections among 480 blinks; then the algorithm's detection sensitivity for blinks is 93% whereas the incorrectly classified blinks is 8 during 213 blinks in the Petterssons paper [28]. Furthermore, the rate for the overall hit of the new system performs was 98% with 8 classes based on linear decoding model, which could be a good effect on the expression of decoding and on-line control. Thus, Usakli et al. classifies 5 classes with nearest neighborhood algorithm, and the classification performance is 92% in real-time [29]. Subsequently the classification performance is increased to 95% [30]. For the response times of system, the time interval of new system is about 500 ms for continuous control output based on eye pursuit movements, and the time delay is less than 1 s, while Masaki Nakanishi employs 600, 700, and 800 ms window length and 100 and 200 ms shift amount. Then, the maximum accuracy of 93.77% is obtained by using 800 ms window and 200 ms shift amount, and there is no mention of the time delay [31]. So the time interval of the new system is shorter than others. Thus the saccade-based systems turned on at a random location 5°, 10°, 20°, or 30° horizontally to the left or right of it in the EOG recording task [14]. So the saccades are strongly limited in their spatial extents to less than a degree of visual angle [32]. All in all, the new system extracts the movement information to predict the target positions in 2D space from EOG. Moreover, whether or not there is a guide curve hints, the new technology is applied to online control a cursor to hit the multiple predefined targets with a higher hit rate than the existing knowledge what we know.

From the view of the control results, the average position error of eye pursuit movements is usually within 1 cm. However, the error of a single individual may be a little larger, which will lead to the control unstable result. Even the error is accumulated with the time increases, so the method cannot be applied to an accurate control system. This also associates with the inherent characteristics of EOG; on the one hand, the EOG is influenced by physiological conditions and itself have great individual difference and instability. On the other hand, the EOG is tightly associated with psychological activity. When the psychological activity is in a bad mood, the effect of control will be worse with the negative psychological activities which reflects easily in eye pursuit movements.

To improve the control accuracy, the EOG data need to be done some postprocessing. Because the decoded eye gazing object positions are not very smooth trajectories which tend to make the controlled cursor shake while moving, it is better to average the decoded cursor positions every 0.5 seconds to be the instant output of the decoding model. The time duration for averaging is so short that the cursor movement would still look continuously. Then, the EOG with high quality is good for online control, but the control may be fail because of the influences of electrode placement, head movements, lighting conditions, sweat, and different sources of artifacts such as EEG and EMG and blinking movements [33]. All subjects are instructed to track the circular cursor with their eyes, no matter whether it is moving or static, with their bodies (especially their heads) still and their eyes blinking naturally. By slightly moving the head, an experienced user can change the relative location between human eyes and the screen and correct the cursor moving error to some degree. Therefore, it needs some amendment and transition in eye pursuit movements to improve the stability of the control result; meanwhile, it is better to make a qualitative control rather than a quantitative control with a high precision. Generally speaking, after the system is calibrated by PLS algorithm, a complete experiment can be carried out by this system for the same subject. But subjects control the system online for a long time (usually more than an hour), or the environment around the subjects take place a series of changes (sweat, the absence of the conductive paste, or the evaporation or being in a bad state), the system needs recalibrating to ensure the accuracy of decoding. When subjects operate error (enter the wrong trajectory) or the actual decoding trajectory is inconsistent with the ideal one evidently, the new system has a dependency on the assistance of HCI and the reset correction of single voluntary blinks to avoid the effect of error accumulation. There is undeniably, the subjects reset correction with blink frequently that lead to slow down the control. Only the subject already is familiar with the eye pursuit movements and the system response, the system can have more effective control.

## 5. Conclusion

This paper proposes a double-threshold-based blink detection method to extract voluntary single blinks and double-blinks. Then, by time series modeling, the system integrates the merits of blinks control and smooth pursuit control to predict target positions well with a continuous linear decoding method in 2D space. Finally, the online calibration and control scheme is carried out to produce free online control without any guide curve hints, and the technology is used to hit multiple predefined on-screen targets successfully with a hit rate of 98%.

Our future work is to focus on the optimization of the online control scheme, and the improvement of control precision. So the technology will be put into use for physically disabled people with still maintain eye movements to help them have very limited peripheral mobility.

## Figures and Tables

**Table 1:** The average values and standard deviations of SNR and *R* for the best, the worst, and the mean of the five participants (mean ± SD).

		Subject 3 (best)		Subject 5 (worst)		Mean (5 subjects)	
		*R*	SNR	*R*	SNR	*R*	SNR
Helix pattern	*X*	0.9934 ± 0.0045	17.38 ± 5.12	0.9919 ± 0.0030	16.75 ± 3.37	0.9914 ± 0.0053	16.87 ± 4.87
	*Y*	0.9708 ± 0.0239	11.90 ± 2.27	0.9028 ± 0.0983	7.58 ± 5.36	0.9312 ± 0.0740	10.39 ± 4.77
Clove pattern	*X*	0.9979 ± 0.0017	18.78 ± 2.86	0.9951 ± 0.0013	14.95 ± 2.76	0.9975 ± 0.0017	20.24 ± 5.44
	*Y*	0.9850 ± 0.0047	12.36 ± 2.63	0.9577 ± 0.0212	9.57 ± 4.08	0.9692 ± 0.0168	10.85 ± 2.47
Stellate pattern	*X*	0.9968 ± 0.0007	15.54 ± 2.87	0.9916 ± 0.0036	15.63 ± 0.58	0.9940 ± 0.0053	16.75 ± 3.31
	*Y*	0.9890 ± 0.0011	14.52 ± 2.48	0.9732 ± 0.0209	10.58 ± 3.18	0.9772 ± 0.0201	13.48 ± 3.84
